# Genetic variation in a member of the laminin gene family affects variation in body composition in *Drosophila *and humans

**DOI:** 10.1186/1471-2156-9-52

**Published:** 2008-08-11

**Authors:** Maria De Luca, Michelle Moses Chambers, Krista Casazza, Kerry H Lok, Gary R Hunter, Barbara A Gower, José R Fernández

**Affiliations:** 1 Department of Nutrition Sciences, School of Health Professions, University of Alabama at Birmingham, 3rd Avenue South, Birmingham, AL 35294-3360, USA; 2 Department of Human Studies, School of Education, University of Alabama at Birmingham, 13th Street South, Birmingham, AL 35294-1250, USA; 3 Clinical Nutrition Research Center, University of Alabama at Birmingham, Birmingham, AL 35294, USA

## Abstract

**Background:**

The objective of the present study was to map candidate loci influencing naturally occurring variation in triacylglycerol (TAG) storage using quantitative complementation procedures in *Drosophila melanogaster*. Based on our results from *Drosophila*, we performed a human population-based association study to investigate the effect of natural variation in *LAMA5 *gene on body composition in humans.

**Results:**

We identified four candidate genes that contributed to differences in TAG storage between two strains of *D. melanogaster*, including *Laminin A *(*LanA*), which is a member of the α subfamily of laminin chains. We confirmed the effects of this gene using a viable *LanA *mutant and showed that female flies homozygous for the mutation had significantly lower TAG storage, body weight, and total protein content than control flies. *Drosophila LanA *is closely related to human *LAMA5 *gene, which maps to the well-replicated obesity-linkage region on chromosome 20q13.2-q13.3. We tested for association between three common single nucleotide polymorphisms (SNPs) in the human *LAMA5 *gene and variation in body composition and lipid profile traits in a cohort of unrelated women of European American (EA) and African American (AA) descent. In both ethnic groups, we found that SNP rs659822 was associated with weight (EA: *P *= 0.008; AA: *P *= 0.05) and lean mass (EA: *P= *0.003; AA: *P *= 0.03). We also found this SNP to be associated with height (*P *= 0.01), total fat mass (*P *= 0.01), and HDL-cholesterol (*P *= 0.003) but only in EA women. Finally, significant associations of SNP rs944895 with serum TAG levels (*P *= 0.02) and HDL-cholesterol (*P *= 0.03) were observed in AA women.

**Conclusion:**

Our results suggest an evolutionarily conserved role of a member of the laminin gene family in contributing to variation in weight and body composition.

## Background

As the prevalence of obesity and its related co-morbidities continue to increase worldwide [1], there is considerable effort being devoted to identify genetic pathways and mechanisms that control fat storage. To gain insights into the genetic basis of natural variation in fat storage, we have used *D. melanogaster *as a model system. Like mammals, insects store fat as TAG in neutral lipid droplets that are accumulated in the fat body, the functional equivalent of both mammalian liver and white adipose tissue. *Drosophila *shares many of the components of TAG biosynthesis, degradation, and regulation with mammals, including many of those implicated in human lipodystrophies, diabetes, and obesity [2]. *D. melanogaster *has proven to be an important model system to identify genetic loci that contribute to variation in quantitative traits, including lipid metabolism [3,4]. In contrast to rodent and human mapping efforts where high-resolution mapping is constrained by intensive labor demands and expense [5], in *Drosophila *the transition from chromosomal regions [quantitative trait loci (QTL)] identified by recombination mapping to candidate genes [quantitative trait genes (QTGs)] is made possible through the use of quantitative complementation (QC) tests with deficiency and mutant stocks [4,6]. This approach has been highly effective for identifying genetic loci within QTL that contribute to variation in several *Drosophila *traits, including low heritability traits such as olfactory behavior and life-span [4,7]. The QC test has been also used in mice to investigate the effect of a mutation of the *Rgs2 *gene on anxiety behaviors [8]. Recently, *Drosophila *deficiency mapping has been greatly enhanced by the release and availability of the DrosDel and Exelixis deficiency stocks in which all deficiencies occur in the same genetic background and have molecularly defined breakpoints [9,10]. The availability of Exelixis *P *and *piggyBac *stocks with single gene insertions all in the same co-isogenic background [11] has also significantly improved our ability to identify positional candidate genes within refined QTL regions.

We previously mapped multiple QTL responsible for natural variation in TAG storage using a population of recombinant inbred (RI) lines derived from two unrelated *Drosophila *strains, *Ore*gon R (*ORE*) and Russian *2b *(*2b*) [3]. In this study we used quantitative deficiency mapping to fine-map two of the TAG QTL, one encompassing the cytological region 27B-30D on chromosome 2 and the other encompassing 63A-65A on chromosome 3. Subsequently, we performed QC tests with single gene mutant stocks to identify four candidate genes influencing TAG levels. One of the genes identified is *uncoupling protein 4c *(*Ucp4c*), which encodes a product involved in uncoupling of oxidative phosphorylation in mitochondria [12]. Notably, two mammalian homologues of *Ucp4c*, ubiquitous UCP2 and skeletal-muscle-specific UCP3, have already been shown to regulate mammalian fatty acid metabolism [13]. In addition, several human population studies have reported a strong association between polymorphic variants in *UCPs *genes and BMI [14]. The remaining three genes are novel candidate genes affecting fat storage: *CG9135*, *CG1399*, and *Laminin A (LanA)*. *CG9135 *and *CG1399 *belong to a family of genes of unknown function [12]. *LanA *encodes a protein belonging to the α subfamily of laminin chains [12]. Laminins are heterotrimeric glycoproteins present in the basement membrane matrix where they play a role in cell-matrix adhesion, migration, growth, and differentiation of various cell types [15]. While in mammals different combinations of five α, four β and three γ chains can assemble into at least 15 diverse laminins [15], *Drosophila *appears to use only one β, oneγ, and two α chains [12]. The *Drosophila *laminin A chain has significant sequence homology with mammalian laminin α5 chain [16]. In humans, laminin α5 is encoded by the *LAMA5 *gene, which spans approximately 78 kb on chromosome 20q13.2-q13.3 [17]. Several genome-wide linkage scans have linked this chromosomal region 20q13.2-q13.3 to variation in body mass index (BMI) and percentage body fat [18]. In addition, QTL affecting body weight and adiposity have been mapped to a region on mouse chromosome 2 that is syntenic with chromosome 20q13.2-q13.3 in humans [18]. Taken together with our results from *Drosophila*, these observations suggested that polymorphisms in *LAMA5 *contribute to natural variation in body weight and adiposity in humans. To explore this hypothesis we examined the association between genetic variants in the human *LAMA5 *gene and phenotypic variation in several anthropometric traits, including those reflecting body composition and lipid profile in a in a cohort of 228 unrelated EA and AA pre-menopausal women. We selected three haplotype-tagging SNPs from the International HapMap project : rs659822 (T > C) in intron 1, rs2297588 (G > A) in intron 51, and rs944895 (T > C) a non-synonymous SNP in exon 68. Our results imply that genetic variation in the *LAMA5 *gene affects variation in human body composition and lipid profile. However, additional genetic work and functional studies will be necessary to identify causal associations.

## Results

### Fine mapping and identification of positional candidate genes for TAG storage in *Drosophila*

**Figure 1:**
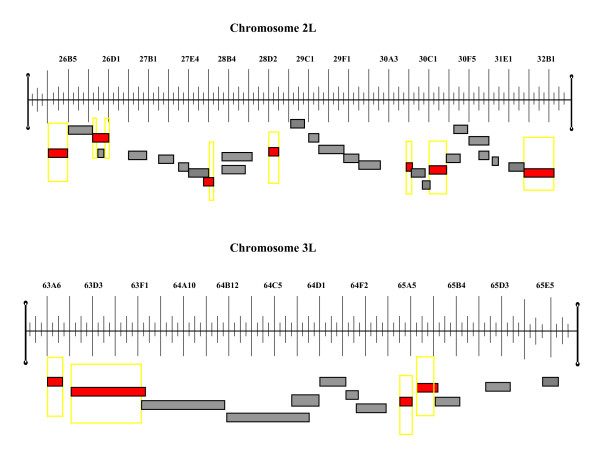
**Quantitative deficiency mapping of *D. melanogaster *TAG QTL**. Long ticks mark sections and short ticks mark subsections of physical maps in the cytological interval 25F5;32B3 on the left (L) arm of chromosome 2 and in the cytological interval 63A6;65E8 on the left arm of chromosome 3. Gray bars represent non-significant deficiencies and red bars correspond to deficiencies with significant failure to complement *ORE *and *2b *QTL for TAG storage. Yellow frames indicate regions where a QTL affecting TAG content between *ORE *and *2b *maps.

We tested the effects of 38 deficiencies that span the QTL intervals at cytological regions 27B-30D and 63A-65A. After Bonferroni corrections for multiple comparisons, seven deficiencies significantly failed to complement the TAG storage phenotypes of *ORE *and *2b *in the 27B-30D QTL region and four deficiencies in the 63A-65A QTL region (Figure 1). The combined data therefore revealed multiple sub-QTL regions each containing at least one gene affecting variation in TAG storage (Figure 1).

To identify candidate genes affecting TAG levels we then performed QC tests using crosses of the *ORE *and *2b *parental strains to mutants of 21 of the genes that map in the refined sub-QTL regions (see Additional File 1: Summary of quantitative complementation tests with mutants of positional candidate genes in *D. melanogaster*). Four of the genes tested showed a quantitative failure to complement, indicating that allelic differences between *ORE *and *2b *strains at these loci contribute to the differences in TAG storage between the two strains: *Ucp4c*, *CG9135*, *CG13993*, and *LanA *(see Additional File 1: Summary of quantitative complementation tests with mutants of positional candidate genes in *D. melanogaster*).

### *Drosophila LanA *influences TAG storage, live weight, and total protein content

**Figure 2:**
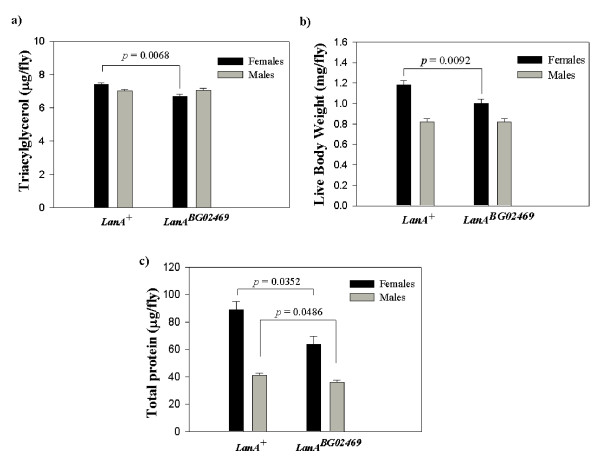
**Effects of *Drosophila LanA *allele on TAG storage, body weight, and total protein content**. Values represent mean ± SEM of TAG storage (panel a), live body weight (panel b), and total protein content (panel c) for *n *= 9 independent replicates of homozygous *LanA*^*BG*02469 ^and wild-type male and female flies.

To independently verify the effect of the *LanA *gene on TAG storage, we measured this trait in flies that were homozygous for the insertional mutation *LanA*^*BG*02469 ^and non-mutant flies from the co-isogenic control line. We also investigated the effects of the insertion on live body weight and total protein content. For male flies, we found no significant difference between mutant and controls for TAG and live body weight (Figure 2a and 2b). However, *LanA*^*BG*02469 ^male flies had slightly reduced total protein content (*P *= 0.0486) compared to controls (Figure 2c). On average, total protein content of *LanA*^*BG*02469 ^males was 10% lower than that of controls. While the effect on males was minimal, the *LanA*^*BG*02469 ^mutation had a dramatic effect on female traits. Females with the *LanA*^*BG*02469 ^mutation had significantly lower TAG storage (*P *= 0.0068), live body weight (*P *= 0.0092), and total protein content (*P *= 0.0352) compared to control flies (Figure 2a–c). The reductions in female TAG storage, live weight, and total protein content relative to control flies were 10%, 16%, and 29%, respectively.

### Human *LAMA5 *variants contribute to variation in anthropometric traits, body composition, and serum lipids

Table 1 summarizes the baseline characteristics of the human cohort stratified by ethnicity. Significant differences in total fat mass (TFM), serum TAG levels, and high density lipoprotein cholesterol (HDL-C) were observed between EA and AA. EA had higher mean TFM and serum TAGs and AA had higher mean HDL-C (Table 1).

**Table 1:** Characteristics of the human subjects by ethnicity

Phenotype (unit of measurement)	European Americans	African Americans
	(*n *= 101)	(*n *= 127)
Age (yr)	34.66 ± 6.0	33.4 ± 5.7
BMI	27.56 ± 2.18	27.50 ± 2.44
Height (cm)	165.6 ± 0.6	163.9 ± 0.6
Weight (kg)	75.67 ± 9.1	73.67 ± 9.0
Total fat mass (kg)	32.14 ± 6.9*	30.29 ± 6.7
Lean tissue mass (kg)	40.22 ± 3.6	39.78 ± 4.4
Triacylglycerol (mg/dl)	115.06 ± 57.3***	67.3 ± 25.6
Total cholesterol (mg/dl)	160.24 ± 31.2	155.86 ± 34.1
HDL-cholesterol (mg/dl)	36.14 ± 9.2***	43.21 ± 10.8
LDL-cholesterol (mg/dl)	101.08 ± 26.7	99.18 ± 31.9

Values are means ± SD. *P *values for difference between European Americans and African Americans are obtained using Student's *t*-test. *p < 0.05 and ***p < 0.001.

The allele and genotype frequencies for each SNP are shown in Table 2. All genotype groups were in Hardy-Weinberg equilibrium. There was no difference in allele or genotype frequencies between EA and AA for SNP rs2297588. However, there was a difference in genotype frequencies between EA and AA for SNP rs944895 and rs659822 (Table 2). These differences remained when the two populations were tested for pair-wise linkage disequilibrium (LD) between SNPs. In both populations rs2297588 was in weak LD with rs659822 (EA: r^2 ^= 0.16; AA: r^2 ^= 0.14) and was more highly lined with rs944895 (EA: r^2 ^= 0.38; AA: r^2 ^= 0.35), whereas rs944895 was associated with rs659822 in the EA population (r^2 ^= 0.37), but not in the AA population.

**Table 2:** Allele and genotype frequencies of *LAMA5 *rs659822, rs2297588, and rs944895 polymorphisms in the study sample

	European Americans						African Americans					
	(*n *= 101)						(*n *= 127)					
	rs659822		rs2297588		rs944895		rs659822		rs2297588		rs944895	

Genotype frequency	TT	0.455^b***^	GG	0.524	TT	0.416^b**^	TT	0.229	GG	0.551	TT	0.260
	TC	0.416	GA	0.446	TC	0.495	TC	0.543	GA	0.417	TC	0.520
	CC	0.129	AA	0.030	CC	0.089	CC	0.228	AA	0.032	CC	0.220
												
Allele frequency	T	0.663^b*^	G	0.748	T	0.664	T	0.501	G	0.760	T	0.520
	C	0.337	A	0.252	C	0.336	C	0.499	A	0.240	C	0.480
												
HWE^a^	0.658		0.117		0.382		0.475		0.149		0.727	

^a^*P *values of Hardy Weinberg equilibrium (HWE) tests. ^b^Comparison between racial groups; *p < 0.05, **p < 0.01, ***p < 0.001.

We next examined whether the SNPs were independently associated with each trait. As significant differences in genotype frequencies were observed for SNP rs944895 and rs659822 between the two populations, we tested the association between SNPs and each trait using the data separately by ethnicity. Age, genetic admixture, and appropriate potential confounding variables were included in the analysis as covariates. While no association was found between SNP rs2297588 and any of the traits (data not shown), we did find significant associations between SNP rs659822 and height, body weight, TFM, and lean tissue mass (LTM) in EA (Table 3) assuming a model of additive effects. When we fit the data to a recessive genetic model we also found a significant association between SNP rs659822 and HDL-C in EA (Table 3). On average, EA women that are homozygous for the C allele had short stature, lower mean body weight, TFM, and LTM than those homozygous for the T allele (Table 3; *P *< 0.05). EA women that were homozygotes for the C allele also had higher levels of HDL-C than those carrying at least one T allele (Table 3; *P *< 0.05). The association between SNP rs659822 and variation in weight and LTM was also observed in AA women (Table 3). In this case, however, AA women homozygous for the SNP rs659822 C allele had *higher *mean weight and LTM than those homozygous for the T allele or heterozygous (Table 3; *P *< 0.05). Finally, significant associations were observed in AA women between alternative alleles at rs944895 and variation in serum TAG levels and HDL-C assuming the additive and dominant models, respectively (Table 3). On average, AA women homozygous for the T allele had lower TAG levels than those homozygous for the C allele and lower HDL-C than those carrying at least one C allele (Table 3; *P *< 0.05).

**Table 3:** Mean ± SEM for anthropometric measures, body composition, and serum lipid profile of study subjects stratified according to *LAMA5 *rs659822 or rs944895 genotype and ethnicity.

	European American				African American			
		
rs659822	C/C	C/T	T/T	P*	C/C	C/T	T/T	*P*
*n*	13	42	46		29	69	29	
BMI	26.8 ± 0.5	27.6 ± 0.3	27.7 ± 0.3	0.1	28.2 ± 0.4	27.2 ± 0.3	27.4 ± 0.4	0.3
Height (cm)	162.6 ± 1.2	164.6 ± 0.8	167.3 ± 1.1	**0.02**	164.9 ± 1.2	164.1 ± 0.8	162.5 ± 1.4	0.2
Weight (kg)	69.8 ± 2.3	74.7 ± 1.3	78.2 ± 1.4	**0.008**	76.5 ± 1.7	73.2 ± 1.1	71.9 ± 1.5	0.05
Total fat mass (kg)	28.8 ± 1.9	31.5 ± 1.0	33.7 ± 1.0	**0.01**	31.3 ± 1.2	30.3 ± 0.9	29.4 ± 1.1	0.3
Lean tissue mass (kg)	37.6 ± 0.8	39.9 ± 0.5	41.2 ± 0.5	**0.003**	41.4 ± 0.8	39.5 ± 0.5	38.7 ± 0.8	**0.03**
Triacylglycerol (mg/dl)	104.9 ± 11.5	120.5 ± 11.4	112.9 ± 6.3	0.8	68.1 ± 4.7	65.8 ± 3.2	70.3 ± 4.3	0.6
Total cholesterol (mg/dl)	153.4 ± 6.3	163.0 ± 4.9	159.6 ± 4.9	0.8	162.3 ± 6.5	150.1 ± 3.8	163.1 ± 6.9	0.6
HDL-cholesterol (mg/dl)	42.4 ± 2.4	36.0 ± 1.6	34.5 ± 1.1	**0.003^a^**	46.1 ± 1.8	41.2 ± 1.3	45.2 ± 2.1	0.07^a^
LDL-cholesterol (mg/dl)	90.0 ± 5.8	102.9 ± 4.1	102.5 ± 4.1	0.5	102.7 ± 6.2	95.8 ± 3.6	103.8 ± 6.4	0.9
								
rs944895								
*n*	9	50	42		28	66	33	
BMI	26.6 ± 0.7	27.5 ± 0.3	27.8 ± 0.3	0.3	27.8 ± 0.4	27.6 ± 0.3	27.1 ± 0.4	0.5
Height (cm)	163.1 ± 1.2	165.6 ± 1.0	166.0 ± 0.9	0.3	163.9 ± 1.4	164.4 ± 0.8	163.1 ± 1.2	0.6
Weight (kg)	70.9 ± 2.8	75.5 ± 1.3	76.9 ± 1.3	0.09	73.5 ± 1.4	74.5 ± 1.2	72.1 ± 1.6	0.7
Total fat mass (kg)	28.2 ± 2.1	31.7 ± 1.0	33.3 ± 1.0	0.1	29.9 ± 1.1	30.7 ± 0.9	29.7 ± 1.2	0.5
Lean tissue mass (kg)	38.5 ± 1.1	40.5 ± 0.6	40.2 ± 0.5	0.7	40.0 ± 0.8	40.2 ± 0.5	38.8 ± 0.9	0.4
Triacylglycerol (mg/dl)	101.8 ± 8.6	117.3 ± 9.2	115.2 ± 8.1	0.9	78.9 ± 5.1	65.7 ± 3.1	60.9 ± 4.0	**0.02**
Total cholesterol (mg/dl)	159.8 ± 7.5	161.2 ± 4.6	159.2 ± 4.9	0.7	156.5 ± 5.6	159.4 ± 4.2	148.3 ± 6.5	0.7
HDL-cholesterol (mg/dl)	41.2 ± 3.1	35.1 ± 1.4	36.3 ± 1.3	0.5^b^	43.4 ± 2.1	45.2 ± 1.4	39.1 ± 1.5	**0.03^b^**
LDL-cholesterol (mg/dl)	98.2 ± 8.6	102.7 ± 3.8	99.8 ± 4.1	0.7	97.4 ± 5.1	101.1 ± 4.0	97.0 ± 6.1	1

* *P *values represent the significance of the comparison among genotypes. *P *values without subscript were calculated assuming additive models. *P *values with subscripts (^a^) and (^b^) were calculated assuming recessive and dominant models, respectively. *P *values significant after permutations tests to correct for multiple comparisons are highlighted in bold case

Except for the association between SNP rs659822 and weight in AA women, all the single-marker associations remained significant at an experiment-wise *P *= 0.05 after allowing for multiple testing by permutation analysis [19]. Pair-wise haplotype-based association analyses did not increase the power of these associations (data not shown).

## Discussion

We performed quantitative deficiency mapping to dissect two previously identified QTL regions influencing variation in TAG storage among a set of RI lines established from two strains of *D. melanogaster*, *ORE *and *2b *[3]. The fine mapping revealed that the two QTL broke down into multiple sub-QTL regions (Fig. 1). This indicates that the number of loci influencing variation in TAG levels among these RI lines is much greater than the number suggested by the initial QTL mapping. This finding is consistent with other studies that have fine-mapped QTL for several quantitative traits in *Drosophila *and mice [4,20,21] and corroborates the complexity of the genetic architecture of quantitative traits.

Several QTL have been associated with BMI, body weight, fat mass, and fat-free mass in human linkage studies [18]. If the complexity observed in model systems turns out to be a common phenomenon also in human traits, then the identification of the genes underlying variation in these traits will remain a challenge. There is increasing evidence that genome-wide association (GWA) studies are a powerful method for identifying genes involved in human complex traits [22]. Taking advantage of the block-like patterns of LD that characterize the human genome [23], these studies rely on the use of hundreds of thousands of "tagging" markers that can capture a significant proportion of the genetic variation and provide power to detect associations. However, one limitation of this approach is that linkage of the markers with variants in a number of genes in the block can make it difficult if not impossible to identify the casual variant affecting the trait. In addition, because of the well-known context dependency of allelic effects of QTL on quantitative traits (e.g. epistasis and genotype by environment interaction) [4], association studies in controlled environments and defined genetic background will potentially allow a more detailed picture of the complexity of the genetic architecture of quantitative traits than that provided by human studies. Studies using *D. melanogaster *and other model systems will continue to play an important role in pinpointing potential candidate loci affecting quantitative traits.

Using QC tests to mutants of positional genes, we identified *Ucp4c*, *CG9135*, *CG13993*, and *LanA *as candidate loci that influence variation in TAG storage between ORE and 2b. Notably, three of the implicated loci, *Ucp4c*, *CG9135*, and *CG13993*, are tightly linked, with *CG9135 *and *CG13993 *being only 9 kb apart [12]. These however represent only a fraction of the genes underlying the QTL effects identified in this study. Together, there are 286 genes currently mapped in the refined QTL regions. Mutant stocks for 99 of these genes are available from the *Drosophila *stock center. Many of these mutants are in different genetic backgrounds making it difficult to distinguish allelic effects on a trait at the tested locus from epistatic effects with genetic background. In this study we therefore chose to focus only on loci with mutations in the same genetic background of their controls. QC tests to all available mutations of the genes mapping within the refined regions are underway.

Our studies in flies further suggest that the effect of *Drosophila LanA *is not limited to TAG storage, but it extends to body weight and whole-body protein content. The observed result on body weight is interesting since three-week old mice homozygous for a hypomorphic mutation in the *LAMA5 *gene, the mammalian homolog of *LanA*, have smaller size than their controls [24]. Here we report that natural variation in this gene may contribute to the underlying variation in these traits in human populations. We identified a significant association between a T/C variant in the human *LAMA 5 *intron 1 (rs659822) and height, body weight, TFM, LTM, and HDL-C in EA women. EA women homozygous for the less frequent variant (CC) on average had lower body weight, TFM, and LTM than those homozygous for the T allele. The effect of SNP rs659822 on weight and LTM was also observed in AA women. In this case, however, AA women that were homozygous for CC at this SNP had higher weight and LTM than women homozygous TT. The opposite effect of rs659822 genotypes on body weight and LTM in the two ethnic groups is intriguing and might be explained by the complexity of the processes that determine variation in these traits, including allelic epistatic interactions within the *LAMA5 *gene and interactions with other genes and with the environment. In this regard it is important to point out that the genotype frequencies of SNP rs659822 were significantly different across these two groups, with the frequency of the genotype CC being significantly lower in EA women than in AA (Table 3). This sensitivity of the allelic effects of SNPs on phenotypic traits has also been observed in disease-marker studies [25] and is implied in QTL studies in plants [26], *Drosophila *[27], and mice [28] that show significant differences in allelic effects on phenotypes depending on the genetic background in which they occur. Lin *et al*. used theoretical modeling to demonstrate that such "flip-flop" associations can occur because the lack of consideration of other genetic loci or environmental factors that influence complex traits [25]. They argue that this is particularly important when a non-causal genetic variant that is linked with the causal polymorphism is investigated [25]. Because genotypes of all polymorphic sites in the *LAMA5 *gene were not determined and SNP rs659822 is located in an intron, it is possible that rs659822 is not itself the causal polymorphism, but is in LD with the true causal polymorphism somewhere else in this gene. In our results SNP rs659822 was in weak LD with both rs2297588 (r^2 ^= 0.16) and rs944895 (r^2 ^= 0.37) in EA and both SNPs were not associated with any of the traits in this ethnic group. This observation suggests that SNP rs659822 is the site with the largest association with the true causal polymorphism. An overview of the pattern of linkage disequilibrium across the *LAMA5 *gene established by the HapMap Project in the CEU population of northern and western European ancestry from Utah showed that SNP rs659822 is in strong LD (r^2 ^= 0.73) with a non-synonymous A to G variant in exon 47 (rs2274934) that could be the responsible polymorphism. Notably, this SNP converts the neutral amino-acid asparagine to the negatively charged amino-acid aspartate in one of the laminin EGF-like domains, which have been suggested to act as signals for cellular growth and differentiation [29]. A change in the amino acid structure of this laminin EGF-like domain might explain our finding that variation in *LAMA5 *associates with a pleiotropic effect on both anthropometric traits and body composition.

We also identified a significant association between a non-synonymous T to C variant in the exon 68 (rs944895) that converts a tryptophan to an arginine in the laminin G (LG)-like 2 domain and variation in serum TAG levels and HDL-C and in AA subjects. AA women homozygous for the less frequent variant (CC) on average had lower serum TAG and HDL-C levels than those homozygous for the T allele. Laminin LG modules have been implicated in interactions with cellular receptors and other extracellular ligands, such as heparan sulfate proteoglycans (HSPGs) [30]. Interestingly, consistent evidence exists that HSPGs play a role in the turnover of lipoproteins, including the uptake of HDL-C in liver [31]. Moreover, cell surface HSPGs contribute to intracellular TAG accumulation in adipocytes [32]. Studies examining the functional effect of rs944895 polymorphism in lipoprotein metabolism will be necessary to understand the mechanisms underlying our findings.

One limitation of our study is that the human association component involved a fairly small sample size and was restricted only to women. This cohort was chosen because measurements of genetic admixture were available for each individual, which allowed us to adjust for ancestry within ethnic groups and, therefore, limit false-positive results [33]. The human data set was also chosen because it provided detailed measurements of body composition for each individual. Clearly, replication of the results in other human cohorts is necessary [34], but the consistency in genetic effects of this member of the laminin gene family in both the fly and humans supports a generally conserved role for this gene in regulating traits reflecting body composition. This is particularly evident for the effect of the gene on lean tissue mass, which was not only observed in both EA and AA women, but also in male and female *Drosophila*.

## Conclusion

Over the past few years, the number of chromosomal regions that contain one or more genes affecting obesity traits in humans and in mammalian models has dramatically increased [18]. Results from our study indicate that *D. melanogaster *may be a good model to pinpoint those genes with evolutionarily conserved effects on body composition that fall within the large chromosomal regions identified in mammalian QTL studies. Our cross-disciplinary genetic study implicates a member of the laminin gene family as a novel candidate gene affecting variation in body composition traits in natural populations. These observations motivate future studies in independent human populations to verify the effects of this gene.

## Methods

### *Drosophila *deficiency and mutant complementation mapping

#### *Drosophila *stocks

Deficiency stocks used for the deficiency complementation mapping were obtained from the Bloomington Drosophila Stock Center . All deficiencies used in our study are from the Exelixis and DrosDel collections that have been generated in co-isogenic *w*^1118 ^backgrounds [9,10].

Mutant stocks were obtained from the Bloomington *Drosophila *Stock Center and from Trudy Mackay at NC State. Except for *LanA*^*BG*02469^, all the mutations are DrosDel and Exelixis *P *and *piggyBac *insertions in the *w*^1118^co-isogenic background. *LanA*^*BG*02469 ^is a hypomorphic mutation generated by the insertion in the *w*^1118^*;Canton S *strain of a *P*-element that is located 339 bp upstream the coding region of the *LanA *gene. Flies were maintained in vials containing 10 ml of standard cornmeal, agar, sugar, and yeast medium at 25°C.

#### Experimental design and phenotypic measurements

We conducted the QC tests with deficiencies and mutations using *ORE *and *2b*, the parental lines used to establish the mapping population for the recombination mapping study [3]. In our experiments, we crossed virgin females from *ORE *and *2b *to males from each deficiency stock and to males from the *w*^1118 ^strain. These crosses produce four possible genotypes: *ORE*/*Deficiency*, *ORE*/*w*^1118 ^and *2b*/*Deficiency*, *2b*/*w*^1118^. We measured four replicate trait values of each genotypic class for each sex using the same experimental design described in [3], with the only exception being the way TAG content was measured. Briefly, we kept each genotypic class of flies in four replicate vials, each containing a group of 10 single-sexed individuals. After 4–5 days, we anesthetized each group of flies and measured live weight to 0.01 mg accuracy with an analytical balance. We then homogenized the flies using the protocol described in. We assayed TAG content spectrophotometrically using a commercially available kit (Sigma-Triglyceride Assay Kit) following the manufacturer's suggested protocol. To account for difference in body weight and total protein content, we used the live weight and total protein content as covariates in the analysis of the data. We measured total proteins for each homogenate using a standard Lowry protein assay.

We conducted the QC test with mutant stocks using the same experimental design described for deficiencies, with the only exception being that nine replicate trait values of each genotypic class were measured for each sex.

### Statistical analyses

All quantitative complementation tests to deficiencies were carried out simultaneously. A quantitative failure of *ORE *and *2b *QTL alleles to complement a deficiency was inferred if the difference in the mean trait value between the *ORE *and *2b *alleles over the deficiency was significantly greater than the difference in the mean trait value of the *ORE *and *2b *alleles over *w*^1118 ^[35]. In a three-way factorial analysis of covariance (ANCOVA), these differences are indicated by a line-by-genotype (*L *× *G*) or line-by-genotype-by-sex (*L *× *G *× *S*) interaction terms according to the model: *y *= *μ + L + G + S + LW + PRO + L × G + L × S + L × G × S + E*, in which *μ *is the overall mean, *L *is the fixed main effect of line (*ORE *or *2b*), *S *is the fixed main effect of sex, *G *is the fixed main effect of genotype (*Def *or *w*^1118^), *LW *and *PRO *are the covariates live body weight and total protein content, and *E *is the error term. A significant *L *× *G *× *S *interaction term is indicative of a sex-specific failure to complement. The deficiencies that showed a significant failure to complement were confirmed by re-testing nine replicate trait values of each genotypic class for each sex. Bonferroni corrections were performed to control for the effect of multiple comparisons.

The data from QC tests with mutations were analyzed for each sex separately using the two-way factorial model of ANCOVA: *y *= *μ + L + G + LW + PRO + L × G + E*, in which *μ *is the overall mean, *L *is the fixed main effect of line (*ORE *or *2b*), *G *is the fixed main effect of genotype (*Def *or *w*^1118^), *LW *and *PRO *are the covariates live body weight and total protein content, and *E *is the error term. We inferred a quantitative failure of *ORE *and *2b *QTL alleles to complement the mutant allele if the *L *× *G *interaction term was significant. In addition, as no significant difference in TAG content was observed between the parental lines *ORE *and *2b*[3], we also considered a significant *L *term as a failure to complement, if the difference between the parental strains was significant in the mutant background but not in the *w*^1118 ^chromosome background [3]. Bonferroni corrections were performed to control for the effect of multiple comparisons.

The statistical analyses were carried out using the SAS GLM procedures (Version 9.0; SAS Institute, 2002, Cary, NC, USA).

### Human study

#### Subjects

A total of 228 European-American (n = 101) and African-American (n = 127) women were evaluated for the human association study. Subjects were participants of two ongoing longitudinal studies on the role of metabolism in the etiology of obesity conducted in AA and EA pre-menopausal women at the University of Alabama at Birmingham. Prior to testing, subjects were maintained in a weight-maintenance state for 4 weeks. During the final 2 weeks, meals were provided through the General Clinical Research Center at UAB to ensure weight stability of less than 1% variation and to maintain daily macronutrient intake in the range of 20–23% fat, 16–23% protein, and 55–64% carbohydrate. Subjects were then admitted as inpatients to the GCRC for 4 days, during the follicular phase of the menstrual cycle. All metabolic testing took place during this inpatient period. At the time of testing, subjects were sedentary (no previous history of exercise training), had a BMI range between 24 – 30 kg/m^2^, were nonsmokers, and were not taking any medication known to alter body composition (including hormones). Race was determined by self-reported African-American or Caucasian ancestry in both parents and grandparents.

The study protocol was approved by the Institutional Review Board for human studies at the University of Alabama at Birmingham. A written informed consent was obtained from all study participants before enrolling in the study.

#### Anthropometrical and serum lipid measurements

Height and body weight were measured in light indoor clothes and without shoes. Blood samples were withdrawn after 12-h overnight fast. Analyses for serum lipids were performed in the Core Laboratory of the General Clinical Research Center and the Clinical Nutrition Research Center (CNRU) at UAB. Total cholesterol, HDL-C, and TAGs were measured with the Ektachem DT II System. With this system, HDL-C is measured after precipitation of low-density lipoprotein cholesterol (LDL-C) and very-low-density lipoprotein cholesterol with dextran sulfate and magnesium chloride. Control sera of low and high substrate concentration are analyzed with each group of samples, and values for these controls must fall within accepted ranges before samples are analyzed. The DT II is calibrated every six months with reagents supplied by the manufacturer. LDL-C was estimated using the Friedewald formula [36].

#### Body composition

Body composition [TFM and LTM] was measured by dual energy X-ray absorptiometry using either a Lunar DPX-L densitometer (LUNAR Radiation Corp., Madison, WI) or a LUNAR Prodigy densitometer in the Department of Nutrition Sciences at UAB. Body composition assessed by these instruments generally differs by a coefficient of variation of 4% or less [37]. Subjects were scanned in light clothing while lying flat on their backs with arms at their sides.

#### Genotyping

To test for associations between genetic variants in human *LAMA5 *and phenotypic traits, we selected three of the human *LAMA5 *SNPs identified by the International HapMap project. Using HapMap data release #21a, we estimated that these three SNPs captured 95.6% of common variation (Minor Allele Frequency >0.05, *n *= 23) at an *r*^2 ^> 0.8 across *LAMA5 *gene. The genotypes of these polymorphisms were determined by Pyrosequencing technology [38] at the CNRU Genetics Core at UAB.

To account for the confounding effects of population stratification, we used estimates of genetic admixture as a covariate in statistical models. The genetic admixture estimates were obtained from the genotyping of ancestry informative markers (AIMs) across the human genome. These AIMs are informative for parental ancestry, defined as those long-separated populations that intermixed during historical periods to produce new admixed populations. Genotyping for the measures of genetic admixture was performed at Prevention Genetics  using the McSNP method and agarose gel electrophoresis, as previously described [39]. Molecular techniques for the allelic identification and methodology for genetic admixture application have been described elsewhere[40,41]. Approximately 100 ancestry informative markers were utilized for the study. Information regarding marker sequences, experimental details, and parental population allele frequencies has been submitted to dbSNP  under the handle PSU-ANTH.

### Data Analyses

We assessed Hardy-Weinberg equilibrium, estimated haplotype frequencies, and r^2 ^linkage disequilibrium coefficients by methods implemented in Arlequin program 3.01 [42]. Allele and genotype frequency comparisons between EA and AA samples were performed by the χ^2 ^test. To test the effect of each genotyped SNP on trait variation, we performed genotypic associations for dominant, additive, and recessive models using linear regression analysis. Age and genetic admixture were used as covariates in all the analyses. As strong correlations have been shown between fat mass and lipid profile [43], lipoprotein levels were additionally adjusted for TFM and TAGs. TAG levels were also adjusted for TFM. Dummy variables were assigned to code the three genotypes in each model. In the additive model, we used 0, 1 and 2 to code for individuals homozygous for the major allele, heterozygous, and homozygous for the minor allele, respectively. In the dominant and recessive models, we used 0 to code for individuals homozygous for the major and minor alleles, respectively, and 1 to code for individuals carrying at least one copy of the other allele. Pair-wise haplotype-based association analyses were also performed. For all regression models, studentized residuals were evaluated for normality and logarithmic transformations of the dependent variable was performed to improve normality. When normality of the residuals was not obtained after transformations, the observations that were above and below three standard deviations were removed from the analyses. To test for significant differences among means according to genotype, data from the final regression model was analyzed by analysis of variances and mean differences assessed by post-hoc Duncan tests at *P *< 0.05. To control for the effect of multiple comparisons, we performed permutation tests (1000 simulations) to generate empirical *P *values under the null hypotheses of no association between genotypes and traits [19]. All the analyses were performed using SAS (Version 9.0; SAS Institute, 2002, Cary, NC, USA).

## Authors' contributions

MDL conceived the study, participated in its design and coordination, carried out the *Drosophila *data analysis, and wrote the manuscript. MMC carried out the *Drosophila *complementation tests. KC carried out the human statistical analyses. JRF participated in the design and coordination of the study and the human statistical analyses. KHL carried out the human genotyping. JRF, BAG, and GRH contributed to design and acquisition of human data. BAG and JRF revised critically the manuscript. All authors read and approved the final manuscript.

## Supplementary Material

Additional file 1 **Summary of quantitative complementation tests with mutants of positional candidate genes in *D. melanogaster***. The table contains a list of all the positional candidate genes and the corresponding mutant alleles analyzed by quantitative complementation tests. In the table are also reported the cytological positions of the candidate genes and the *P *values for Line and Line × Genotype effects of two-way factorial ANOVAs (see text for further explanation). Click here for file
