# Supplementary material for: Genetic variation in a member of the laminin gene family affects variation in body composition in Drosophila and humans
Source: BMC Genet. 2008 Aug 11;9:52. doi: 10.1186/1471-2156-9-52 (PMC2533007; doi:10.1186/1471-2156-9-52)
Supplement: Additional file 1 — Summary of quantitative complementation tests with mutants of positional candidate genes in D. melanogaster. The table contains a list of all the positional candidate genes and the corresponding mutant alleles analyzed by quantitative complementation tests. In the table are also reported the cytological positions of the candidate genes and the P values for Line and Line × Genotype effects of two-way factorial ANOVAs (see text for further explanation). [file 1471-2156-9-52-S1.doc]

**Additional File 1 - Summary of quantitative complementation tests with mutants of positional candidate genes in *D. melanogaster.***

|  |  |  | Males | |  | Females | |
| --- | --- | --- | --- | --- | --- | --- | --- |
| Gene | Alleles | Cytological position | L | L X G |  | L | L XG |
|  |  |  |  |  |  |  |  |
| *blue cheese* | *w^1118^; PBac{RB}bchs^e00833^* | 26A1 | 0.3323 | 0.8800 |  | 0.0762 | 0.7264 |
| *dissatisfaction* | *w^1118^; PBac{WH}dsf^f00109^* | 26A1 | 0.1211 | 0.9106 |  | 0.6814 | 0.5572 |
| *Uncoupling protein 4C* | *w^1118^; PBac{RB}Ucp4C^e03988^* | 26A4-5 | 0.5243 | 0.0334^a^ |  | 0.0247 | **0.0015** |
| *CG9135* | *w^1118^; PBac{WH}CG135^f03307^* | 26B3 | 0.0351 | **0.0007** |  | **0.0011** | 0.1668 |
| *CG13993* | *w^1118^; P{EP}CG13993^EP570^* | 26B4 | 0.7292 | 0.1997 |  | **<.0001** | 0.0129 |
| *Mediator complex subunit 20* | *w^1118^; PBac{WH}MED20^f00955^* | 28E1 | 0.9345 | 0.5661 |  | 0.2228 | 0.8027 |
| *CG8552* | *w^1118^; PBac{WH}CG8552^f04269^* | 28E9 | 0.1884 | 0.9437 |  | 0.0691 | 0.2035 |
| *Thioredoxin 2* | *w^1118^; P{GT1}Trx-2^BG02804^* | 30C1 | 0.8046 | 0.0566 |  | 0.4409 | 0.9812 |
| *cAMP-dependent protein kinase* | *w^1118^; P{GT1}Pka-C1^BG02142^* | 30C5 | 0.5139 | 0.0994 |  | 0.7720 | 0.2529 |
| *Nckx30C* | *w^1118^; PBac{RB}Nckx30C^e00401^* | 30C6-7 | 0.1255 | 0.4583 |  | 0.4639 | 0.9637 |
| *uninitiated* | *w^1118^; P{EP}und^EP424^* | 30C7 | 0.0087^a^ | 0.6916 |  | 0.5539 | 0.1060 |
| *Trim9* | *w^1118^;PBac{WH}Trim9^f001521^* | 31F5-32A2 | 0.5898 | 0.3354 |  | 0.1771 | 0.2986 |
| *CG31871* | *w^1118^; PBac{WH}CG31871^f02763^* | 32A3 | 0.1558 | 0.8522 |  | 0.5482 | 0.5430 |
| *Lipase 2* | *w^1118^; PBac{WH}Lip2^f06907^* | 32A4 | 0.1487 | 0.9610 |  | 0.4411 | 0.0046 |

| *CG17124* | *w^1118^; PBac{PB}CG17124^c01575^* | | 32A5 | 0.3528 | 0.8082 |  | 0.7454 | 0.4723 |
| --- | --- | --- | --- | --- | --- | --- | --- | --- |
| *CG6724* | | *w^1118^; PBac{RB}CG6724^e02149^* | 32A5 | 0.1182 | 0.8825 |  | 0.5412 | 0.8640 |
| *CG6729* | | *w^1118^; PBac{RB}CG6729^e04960^* | 32A5 | 0.3533 | 0.1110 |  | 0.3380 | 0.0937 |
| *CG6750* | | *w^1118^; PBac{RB}CG6750^e02662^* | 32A5 | 0.3148 | 0.9095 |  | 0.5615 | 0.1180 |
| *Nitric oxide synthase* | | *w^1118^; PBac{WH}Nos^f024697^* | 32B1 | 0.0010^a^ | 0.7823 |  | 0.3794 | 0.2679 |
| *porin* | | *w^1118^; PBac{WH}porin^f03616^* | 32B1 | 0.2296 | 0.1111 |  | 0.0855 | 0.6662 |
| *Laminin A* [65A8-65A9](http://flybase.bio.indiana.edu/.bin/fbidq.html?content=maploc&FBgn0002526) 0.0813 0.0114 0.4442 <.0001 0.0131 0.8913 0.0847 | | *w^1118^;P{GT1}LanA*[*^BG02469^*](http://flybase.bio.indiana.edu/.bin/fbidq.html?FBti0018116) | 65A8-A9 | 0.0839 | **0.0015** |  | 0.0935 | 0.5140 |

^a^ Although statistically significant, the difference between tester genotypes is greater than between deficiency genotypes (see text for explanation). *P* values for Line (L) and Line×Genotype (L×G) effects significant after Bonferroni corrections for multiple comparisons are highlighted in bold case.
